# Anti-Müllerian hormone (AMH) as a predictor of antral follicle population in heifers

**DOI:** 10.21451/1984-3143-2017-AR887

**Published:** 2018-08-16

**Authors:** Christopher Júnior Tavares Cardoso, Jair Sábio de Oliveira, Henrique Kischel, Wilian Aparecido Leite da Silva, Elielton Dias da Silva Arruda, Mirela Brochado Souza-Cáceres, Fernanda Amarante Mendes de Oliveira, Ériklis Nogueira, Guilherme de Paula Nogueira, Fabiana de Andrade Melo-Sterza

**Affiliations:** 1 , , .; 2 Veterinary Medicine, Biomedical Sciences Faculty of Cacoal, Cacoal, RO, .; 3 Laboratory of Animal Reproduction Biotechnologies, State University of Mato Grosso do Sul, Aquidauana, MS, .; 4 , , .; 5 Federal Institute of Education, Science and Technology of Amazonas, Tabatinga, AM, .; 6 Embrapa Pantanal, Corumbá, MS, .; 7 State University of São Paulo, Araçatuba, SP, .

**Keywords:** bovine, follicles, selection donors

## Abstract

Antral follicular population (AFP) and anti-Müllerian hormone (AMH) concentration are strongly associated. Thus, analyzing AMH concentration is a reliable method to predict animals with high AFP, an important feature to select donor cows for embryos and oocytes. However, not much is known about this parameter in heifers, particularly in crossbred animals. The aim of this study was to assess AFP in heifers and cows and its relation to serum AMH levels. *Bos taurus indicus* cows (36–60-months-old; n = 30) and heifers (12–14-months-old; n = 17) of the same breed were evaluated. A single procedure of follicular counting was performed by ultrasound for each animal. Random sampling was performed to analyze serum AMH concentration. Cows showed higher AFP than heifers; nonetheless, plasma AMH concentration did not differ between the categories. A high correlation of AFP with plasma AMH concentration was observed in Girolando heifers. Thus, it is suggested that AMH concentration could be a reliable and less invasive method for selecting heifers with high AFP.

## Introduction

Taking advantage of the rusticity of the Gir breed and the dairy productivity of the Holstein breed, Girolando cows are currently the main breed for milk production in Brazil, comprising around 80% of the dairy cattle in the country ( Borges *et al*., 2012 ). To improve dairy production, selection of females with high productivity, better reproductive efficiency, and/or better competence to respond to reproduction biotechnologies is essential.

The first calving age is approximately 35 months for ¾ Holstein × ¼ Gir cows, ( Silva *et al*., 2014 ), however a high variability among herds (lineages) has been observed. Taking that into account, strategies are needed to reduce the age for first calving and to select animals with higher genetic potential to attain the desired traits.

Phenotypic traits linked to infertility are associated with low ovarian antral follicle population (AFP) in *B. taurus* ( Ireland *et al*., 2011 ). Nevertheless, the relationship between AFP and fertility has not been observed in *B. indicus* (Nellore) and crossbred beef cows ( Baruselli *et al*., 2015 ; Morotti *et al*., 2015 ; Silva-Santos *et al*., 2014a ). The number of recruited follicles in each follicular wave is highly variable between individuals; however, a high level of individual repeatability is observed ( Evans *et al*., 2010 ; Oliveira Junior *et al*., 2015; Batista *et al*., 2014 ; Gobikrushanth *et al*., 2017 ). High correlation of AFP with *in vivo* and *in vitro* embryo production may be used as an auxiliary tool to select donor cows for embryos and oocytes ( Silva-Santos *et al*., 2014a ; Baruselli *et al*., 2015 ; Ghanem *et al*., 2016 ).

AFP is positively correlated with anti-Müllerian hormone (AMH) ( Baldrighi *et al*., 2014 ; Guerreiro *et al*., 2014 ). The glycoprotein AMH, which belongs to the transforming growth factor (TGF)-ß family, is only expressed in the gonads ( Cate *et al*., 1986 ) and is correlated to ovarian follicular development ( Visser *et al*., 2007 ). AMH expression is observed in granulosa cells of growing preantral and antral follicles ( Rico *et al*., 2011 ), and it is described as a premature modulator of follicular growth by controlling premature depletion of the follicular reserve in ovaries ( Monniaux *et al*., 2012 ; Durlinger *et al*., 2002 ). Intrafollicular AMH expression increases until the size of the follicle is 5 mm in cows ( Rico *et al*., 2011 ) and 4 mm in humans ( Weenen *et al*., 2004 ) and then starts decreasing in larger antral follicles ( Monniaux *et al*., 2008 ).

Furthermore, the positive association between AMH and total number of follicles has been described in the ovaries of mice ( Durlinger *et al*., 2002 ), women ( Fanchin *et al*., 2003 ), and bovines (*B. taurus* and *B. indicus*) ( Guerreiro *et al*., 2014 ).

In cows, *B. indicus* is superior to *B. taurus* in terms of AFP and plasma AMH concentration ( Batista *et al*., 2014 ). Considering the strong association between AFP and plasma AMH concentration, analyzing AMH concentration is a reliable method to predict AFP ( Ireland *et al*., 2011 ; Rico *et al*. 2011 ).

Plasma AMH concentration and ovarian follicular population in heifers has been studied in Nellore and Holstein pubertal heifers ( Batista *et al*., 2014 ), but not much is known about crossbred pre-pubertal dairy cattle. Understanding the correlation between plasma AMH concentration and AFP in heifers can validate a methodology for premature selection of heifers with high AFP.

Thus, the aim of this study was to evaluate the correlation between serum AMH plasma and AFP in crossbred dairy heifers and cows.

## Material and methods

### Animals and Experimental Design

The study was performed at the State University of Mato Grosso do Sul. Animals from three different herds were used. Girolando breed cows (¾ *B. taurus* × ¼ *B indicus*), 36–60-months-old (n = 30), and heifers, 12–14-months-old (n = 17), were used. None of the heifers exhibited corpus luteum or ovarian follicles above 8 mm at the time of ultrasonography analyses, indicating a high probability of being pre-pubertal. All animals showed a body score condition that ranged from 2.5 to 3.5 (on a scale of 1–5) ( Houghton *et al*., 1990 ) and were maintained in a grazing system with *ad libitum* access to water and minerals. They were neither pregnant nor lactating. All procedures were approved by the Committee of Ethic and Animal Use of the State University of Mato Grosso do Sul, Aquidauana, MS (Protocol CEUA-UEMS 021-2013).

### Ultrasonographic evaluations

A single procedure of follicular counting was performed for each experimental animal. Transvaginal ultrasonography was performed using ultrasound equipment attached to a 7.5-MHz micro-convex transducer (Aquila^®^, Pie Medical, Maastricht, The Netherlands). Before each procedure, feces were removed from the rectum and the perianal area was washed using tap water. Follicles of ≥3 mm in diameter in both ovaries were counted to characterize AFP. In order to count the follicles, the operator promoted a slow rotation of about 180° to make sure all follicles were counted at once. Immediately after counting with the help of a “cineloop” (an instrument of the ultrasound that records images in few seconds), AFP was checked. The same individual performed the counting procedure in all animals.

### Blood collection and hormonal analysis

Of the 47 evaluated animals, serum samples from 11 cows and seven heifers were subjected to the AMH quantification. Blood samples were collected by the venipuncture flow method on the day of AFP counting, were immediately placed in an icebox, and then RT centrifuged at 3000 rpm for 10 min to allow separation of the serum. After that, samples were frozen at −20°C until further analysis. Plasma AMH concentration was assessed using a kit for bovine AMH (ELISA AL-114, Ansh Labs, Webster, TX, USA), and the concentration was expressed as ng/mL. The inter-assay coefficient of variation ranged from 0.28 to 3.15. All assays were performed at the Animal Endocrinology Laboratory of the Paulista State University (UNESP), Araçatuba, SP.

### Statistical Analyses

All data were assessed using the Statistical Analysis System (SAS), version 9.3 and were represented as means ± SD (standard deviation), except for correlation. Follicular population was analyzed using the PROC MIXED option, and Pearson’s correlation was determined using the PROC CORR option of SAS 9.3. The data were assessed according to the number of follicles, and AMH quantification was performed to determine its effect in the animal category. For all analyses, P ≤ 0.05 was considered as significant.

## Results

The number of antral ovarian follicles observed through transvaginal ultrasonography was higher in cows (P < 0.05) than that in heifers. AFP ranged from 5 to 70 follicles in cows and from 10 to 31 follicles in heifers. However, plasma AMH concentration did not vary (P = 0.19) between the categories ( Table 1 ).

**Table 1:** Number of follicles greater than 3 mm and plasma AMH concentration in Girolando cows and heifers (Means ± SD)

	Cows (n)	Heifers (n)	Value P*
Number of follicles	25,93 ± 12,6 (30)	19,23 ± 5,34 (17)	0,05
AMH (ng/mL)	0,34 ± 0,17 (11)	0,59 ± 0,4 (7)	0,19

* Significant values at P ≤ 0.05.

**Figure 1:**
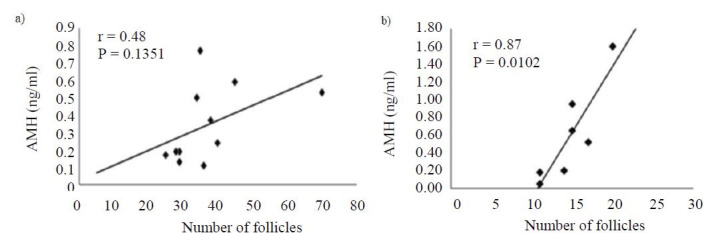
Correlation of plasma AMH concentration and antral follicle ovarian population of cows (A) and Girolando heifers (B).

A high correlation between AFP and AMH concentration (r = 0.87; P = 0.0102) was observed in heifers; however, this correlation was not observed in cows (r = 0.48; P = 0.1351; Figure 1 ).

## Discussion

The present study showed a remarkable difference in AFP between heifers and cows. Nonetheless, no difference was observed in plasma AMH concentration between both categories of Girolando breed.

AMH is an excellent endocrine marker of small antral follicle, which is the direct target of ovarian stimulatory treatments. Plasma AMH concentration before superovulation varies among animals and is positively correlated to the number of ovulations and transferable embryos produced ( Monniaux *et al*., 2010 ; Rico *et al*., 2009 ).

Animals with higher AFP have an increased probability to produce higher numbers of *in vitro* embryos ( Silva-Santos *et al*., 2014b ), thereby propelling the application of this information as a selection trait. Furthermore, high variability in AFP among individuals ( Silva-Santos *et al*., 2014b ) and among distinct bovine breeds has been well-documented ( Rodrigues *et al*., 2015 ; Oliveira Junior *et al*., 2015; Batista *et al*., 2014 ; Guerreiro *et al*., 2014 ; Rico *et al*., 2011 ), indicating that AFP is an important trait for animal selection.

In crossbred beef cows and heifers, AFP is correlated to birth weight and age; an increase in AFP is observed until five years of age, after which AFP decreases ( Cushman *et al*., 2009 ). In consonance with these data, the present study attained higher AFP in cows than that in heifers of the same breed. However, some previous results do not conform to this observation and did not observe any variation in AFP between young and adult Braford heifers ( Silva-Santos *et al*., 2014b ). This divergence between breeds may be due to variations within the individuals of different breeds, lineages, or selective pressure applied in a particular herd.

High plasma AMH concentration is positively associated with the total number of follicles in the ovaries of mice ( Durlinger *et al*., 2002 ) women ( Fanchin *et al*., 2003 ), and bovines ( Batista *et al*., 2014 ). Superior AFP (Holstein cows, 25 follicles; Nellore, 47 follicles) and plasma AMH concentration (Holstein cows, 0.3 ng/mL; Nellore, 0.97 ng/mL) have been observed in zebu heifers compared to those in taurine heifers ( Batista *et al*., 2014 ). Moreover, higher plasma AMH concentration in Nellore cows (2.3 ng/mL) compared to that in Holstein (0.4 ng/mL) has been observed ( Guerreiro *et al*., 2014 ). In the present study, plasma AMH concentration was 0.34 ± 0.17 ng/mL, which is close to that observed for taurine breed.

It is important to note that the animals used in this assay were crossbreeds (¾ *B taurus* × ¼ *B. indicus*); thus, it will be necessary to verify whether the trait increases based on the crossing performed.

Several studies in women ( Fanchin *et al*., 2003 ) and bovines ( Guerreiro *et al*., 2014 ) demonstrated no variation in plasma AMH concentration across the lifespan, particularly between young and adult individuals.

Increasing reproductive biotechnological methods have been performed in young heifers. Recently, Ovum Pick-Up (OPU) has been performed in Holstein calves ( Bayeux *et al*., 2016 ). Despite the difficulty in performing ultrasound examinations in young heifers and considering animal welfare, our results stated that a single plasma AMH analysis could help in selecting heifers with higher AFP that would probably better respond to OPU. The cost of AMH quantification is still the largest limitation of this technique; however, an increased demand could change this situation.

In conclusion, Girolando (¾ Hosltein × ¼ Gir) cows (24–60-months-old) have higher AFP than heifers (12–14-months-old); however, plasma AMH concentration did not differ between the categories. High correlation of AFP and plasma AMH concentration was observed in Girolando heifers. Thus, it is suggested that AMH quantification can predict AFP in Girolando heifers.
